# The Bioaccumulation, Fractionation and Health Risk of Rare Earth Elements in Wild Fish of Guangzhou City, China

**DOI:** 10.3390/ani14243567

**Published:** 2024-12-10

**Authors:** Xiongyi Miao, Xueqin Wei, Xiqian Zhao, Yupei Hao, Wei Bao

**Affiliations:** 1 School of Geography and Environmental Science & School of Karst Science, Guizhou Normal University, Guiyang 550001, China; miaoxy88@126.com (X.M.); weixueqin2024@gznu.edu.cn (X.W.); zhaoxiqian@gznu.edu.cn (X.Z.); 2 Department of Modern Engineering, Anshun Technical College, Anshun 561000, China; 3 Yunan Provincial Bureau of Geology and Mineral Exploration and Development Center Laboratory, Kunming & Ministry of Natural and Resources Kunming Mineral Resource Supervision Inspecting Center, Kunming 650217, China; 4 Institute for Ecological Research and Pollution Control of Plateau Lakes, School of Ecology and Environmental Science, Yunnan University, Kunming 650500, China; 5 College of Environmental Science and Engineering, Ocean University of China, Qingdao 266100, China

**Keywords:** rare earth elements, wild fish, bioaccumulation, health risk assessment, Guangzhou city

## Abstract

In this study, the total content of REEs ranged from 1.32 to 67.74 μg/kg, with a predominant presence of light REEs. The ΔEu and ΔCe values, which exceeded and approached 1, respectively, indicated positive Eu anomalies and low Ce anomalies. Wild fish were categorized into high-, medium-, and low-REEs-bioaccumulation groups using cluster analysis. Higher LRs/HRs and ΔEu values, coupled with lower ΔCe values, in fish from the high-bioaccumulation group suggested that increased bioaccumulation mitigated fractionation. Omnivorous fish with higher REEs levels and lower LRs/HRs indicated broader feeding sources may enhance REE bioaccumulation and diminish fractionation. Elevated REEs concentrations and LRs/HRs in demersal fish highlighted a preferential accumulation of light REEs in the benthic environment. Smaller fish with higher REEs levels but lower LRs/HRs were likely associated with complex feeding sources. Regression analysis revealed that fish with lengths and weights of less than 18 cm and 130 g, respectively, were more susceptible to REEs bioaccumulation. Despite higher ADI values indicating a greater risk for children and *Pelteobagrus fulvidraco*, all ADI values within 70 μg/(kg·d) suggested that fish consumption poses no risk. This study confirmed that the fractionation of REEs in fish can be used to trace their bioconversion.

## Simple Summary

The limited understanding of the fractionation of rare earth elements (REEs) in fish has hindered the effective use of biomonitoring to assess REEs contamination in aquatic environments. Guangzhou City, which faces widespread emissions of REEs, urgently needs biomonitoring of REEs. Therefore, this study took Guangzhou City as a case study and conducted wild fish collections to investigate the bioaccumulation and fractionation of REEs. Additionally, the associated health risks were evaluated to guide fish consuming. This study confirmed the different REEs bioaccumulation and fractionation among 11 fish species. The bioaccumulation of REEs was found to be a process that could mitigate REEs fractionation. The bioaccumulation and fractionation of REEs among fish with feeding behaviors and living habitats were determined to be specific, so that REEs bioaccumulation and fractionation could be used for tracing the environmental behaviors of fish. Food sources should be treated as the critical regulator in varying REEs bioaccumulation and differentiation. The overall low content of REEs in wild fish shows their consumption should not pose a relevant risk.

## 1. Introduction

Rare earth elements (REEs) constitute a distinct group of trace elements, encompassing lanthanum (La; Z = 57) to lutetium (Lu; Z = 71) and yttrium (Y; Z = 39), totaling 15 in number [1]. These elements are lithophilic, exhibiting similar electronic configurations and a stable +3 oxidation state [2]. Their homogeneous nature in the environment stems from their comparable physical and chemical properties [3]. Traditionally, REEs are categorized into light rare earth elements (LREEs), spanning from La to Eu, and heavy rare earth elements (HREEs), ranging from Tb to Lu, inclusive of Y [4]. The electronic, magnetic, optical, and catalytic properties of REEs find extensive applications in advanced technology, the chemical industry, and medical fields [5]. The recent surge in the consumption of REEs across diverse modern applications has significantly escalated their release into aquatic environments, particularly rivers [6,7,8], thereby emerging as contaminants in aquatic biotas [9].

Biomonitoring of contaminants plays a pivotal role in guiding pollution control in aquatic environments [10,11]. Extensive research has been conducted on the bioaccumulation of contaminants in fish, serving as a foundation for global biomonitoring efforts [12,13,14]. However, these studies have primarily focused on traditional contaminants such as heavy metals [15], polycyclic aromatic hydrocarbons [16], and microplastics [17], with less attention paid to emerging contaminants, particularly rare earth elements (REEs). The lack of concern over REEs has hindered the development of biological monitoring for these substances, despite their increasing global usage necessitating such monitoring. Previous studies have emphasized that the selection of organisms for environmental monitoring is closely related to their bioaccumulation of contaminants [13,18], making the determination of REEs bioaccumulation in fish crucial for establishing their effective biomonitoring. Moreover, fish are among the most consumed aquatic products worldwide, providing consumers with abundant protein, unsaturated fatty acids (DHA and taurine), and trace elements [10,19]. However, the risks associated with consuming contaminated fish cannot be overlooked. Previous research has shown that these risks are primarily linked to the ingestion of contaminated fish, prompting numerous studies to propose safer fish consumption practices [15,20,21]. Yet these investigations have primarily focused on heavy metals in fish, limiting the comprehensiveness of health risk assessments for fish consumption. As a significant component of trace elements, the impact of REEs on the health risks associated with fish consumption should not be neglected. Therefore, enhancing the investigation of REE bioaccumulation in fish will improve the comprehensiveness of health risk assessments for fish consumption.

Guangzhou City stands as one of the most densely populated industrial hubs in southern China, nestled in the heart of the Pearl River Delta. The rapid industrialization and urbanization of this city have significantly elevated its wastewater discharge levels [22]. The widespread use of REEs in automobile manufacturing, microelectronic device production, alloy material fabrication, and other high-tech industries has elevated the content of REE in discharged wastewaters [23,24], which has increased the aggregation of REEs in the Pearl River [25,26]. However, nothing has been done to ascertain the bioconversion and ecological effects of these elements. To strengthen the identification of REEs contamination, it is crucial to conduct comprehensive investigations into their bioaccumulation. Furthermore, Guangzhou City boasts a well-developed water system, making it a popular destination for residents engaged in wild fish collecting [15]. As these fish are primarily collected for personal consumption, the unregulated collection and consumption of wild fish have inadvertently heightened the risk of REEs exposure for public health in Guangzhou. Therefore, it is imperative to consider the bioaccumulation, fractionation, and health risks associated with REEs in this city. With this in mind, the present study aims to achieve the following: (1) investigate the bioaccumulation of REEs in 11 dominant species of wild fish collected from Guangzhou City, (2) determine the impacts of feeding behaviors, living habitats, and fish size on the bioaccumulation and fractionation of REEs within these species, (3) evaluate the health risks associated with REEs in wild fish consumed in Guangzhou City. The investigation of REEs bioaccumulation in wild fish in Guangzhou City will not only provide valuable guidance for safe fish consumption but also promote environmental monitoring, control, and governance efforts in the river.

## 2. Materials and Methods

### 2.1. Description of the Study Area and Fish Collection

**Figure 1:**
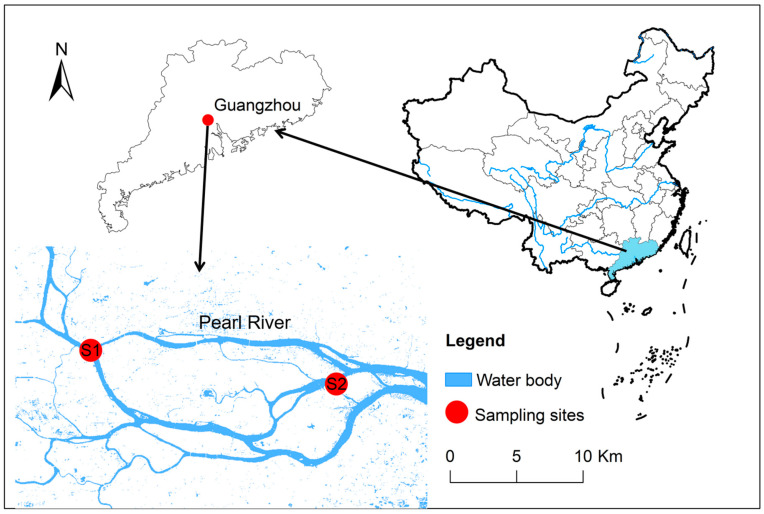
Sampling location of wild fish in Guangzhou City.

Guangzhou City, situated in the southern Chinese province of Guangdong, is home to the vital surface runoff of the Pearl River [27]. This river traverses the entire city, stretching 82.55 km before merging into the South China Sea, covering an area of 38.9 square kilometers [28]. The wastewater emissions of this city amount to a staggering 200 million tons per day [29,30], yet adequate treatment often fails to remove emerging pollutants, particularly for REEs, thereby exacerbating their ecological impact on the Pearl River. Given the dense concentration of high-tech industries and unregulated wild fish collection, primarily in urban areas, two sampling sites (S1 and S2) with significant fish angling activities were identified within Guangzhou City (Figure 1). S1 and S2 are located upstream and downstream of the Pearl River in Guangzhou City section, respectively. Between 10 and 18 July 2021, wild fish samples were directly obtained from anglers at each site. Fish smaller than 10 cm in length and 20 g in weight were excluded from the study due to their limited consumption by the public. Collectively, 137 wild fish belonging to 11 species were gathered from S1 and S2. The species, size, and location of each fish were recorded immediately after collection, with detailed descriptions provided in Table 1. All fish were stored and transported in self-sealing polyethylene bags at temperatures below −20 °C.

### 2.2. Sample Preparation and Analysis

The species, length, and weight of fish were determined before their autopsy. As the most consumed part of fish, 50–100 g flesh from the back muscles was only collected after autopsy. For the same species, several fish with similar length and weight were combined into one to meet the requirement of testing weight. The collected flesh was washed with MilliQ water, then freeze-dried for 72 h to a constant weight at −80 °C. All samples were ground into a powder for further digestion. Then, 0.3 g powder samples were digested with 10 mL HNO_3_ (68%) and 2.0 mL H_2_O_2_ (30%) via microwave digestion at 140 °C for 6 h. The content of 15 REEs in fish, namely lanthanum (La; Z = 57) to lutetium (Lu; Z = 71) and yttrium (Y; Z = 39), were determined using ICP–MS (Thermo X, ThermoFisher, Waltham, MA, USA) with a standard calibration curve and internal standards (10 ppb) Sc, In, Rh, and Ru for mass correction. The O_2_ and KED modes were used for REEs analysis in the ICP–MS, the relevant operating conditions of which can be found in Table 2. The detected REEs consisted of 6 LREEs and 9 HREEs. Among them, the concentration of Tm and Lu were extremely low in fish, both of which were below the detection limits and showing no detection. Therefore, only 13 REEs were obtained in this study. The moisture rate of fish was obtained with the weight before and after freeze-drying. These moisture data were used to recover the wet weight of REEs in fish. The GBW10018 standard substance for fish [31], sourced from the Chinese Academy of Sciences, served as the certified reference material. This, along with the reagent blank and duplicates, was prepared using the previously mentioned procedures, adhering to the standard sample preparation guidelines. To maintain rigorous quality assurance and control, a blank and a standard were incorporated into every batch of ten samples during digestion. Each sample was subjected to three measurements to guarantee a relative standard deviation below 5%. Furthermore, the individually calculated recovery rates for each element fell within the acceptable range of 95–105%, confirming adherence to the study’s established QA/QC protocols.

### 2.3. Assessment Method

#### 2.3.1. The Parameters of Rare Earth Elements

ΔCe and ΔEu are commonly used to expressed the fractionation of REEs [32] and were calculated with the standardization of chondrites:

(1) $$\Delta Ce=\frac{\frac{Ce_{\mathrm{sample}}}{Ce_{\mathrm{reference}}}}{\sqrt{\frac{La_{\mathrm{sample}}}{La_{\mathrm{reference}}}\times \frac{Pr_{\mathrm{sample}}}{Pr_{\mathrm{reference}}}}}$$

(2) $$\Delta Eu=\frac{\frac{Eu_{\mathrm{sample}}}{Eu_{\mathrm{reference}}}}{\sqrt{\frac{Sm_{\mathrm{sample}}}{Sm_{\mathrm{reference}}}\times \frac{Gd_{\mathrm{sample}}}{Gd_{\mathrm{reference}}}}}$$

Sample and reference in Equations (1) and (2) express the measured values in fish and their corresponding values in chondrites. Values of ΔEu and ΔCe beyond (1) suggested a positive anomaly, while a negative anomaly could be confirmed by values below (1).

#### 2.3.2. The Evaluation of Health Risk in Fish

The daily intake of REEs via fish consumption (ADI) was employed in evaluating health risk, which was proposed previously by USEPA [33].

(3) $$\mathrm{ADI}=\frac{C\times GW\times EF\times ED}{BW\times AT}$$

In Equation (3), C is the content of REEs in wet weight (μg/kg), GW expresses the daily intake of fish (g/d), EF and ED are exposure frequency (days/year) and duration (year), respectively, while BW and AT represent the body weight (kg) and average time of exposure (day/year). The detail of these parameters can be found in Table 3.

### 2.4. Statistical Analysis

The data analysis was performed with Excel 2010. The tables and figures were created with OriginPro 8 and Coreldraw X7. Cluster analysis was used for grouping the bioaccumulation of REEs between species, while correlation and regression analysis were applied in gauging the impacts of fish size on REEs bioaccumulation and fractionation. All of these analyses were conducted using SPSS 22. All figures were completed with OriginPro 8.

## 3. Results and Discussion

### 3.1. The Bioaccumulation of REEs in Fish

Thirteen target rare earth elements (REEs) were detected in all fish samples from Guangzhou City, with the exception of Tm and Lu, which had extremely low contents below the detection limits and were therefore excluded from the study. The total content of REEs ranged from 1.32 to 67.74 μg/kg, with an average of 13.14 μg/kg. Specifically, the total content of light rare earth elements (LREEs) ranged from 1.09 to 53.79 μg/kg, averaging 10.64 μg/kg, while the total content of heavy rare earth elements (HREEs) ranged from 0.22 to 13.95 μg/kg, averaging 2.50 μg/kg (as shown in Table 4). Notably, the total content of LREEs was significantly higher than that of HREEs. Among the individual REEs, the mean content decreased in the following order: Ce > La > Nd > Y > Pr > Sm > Gd > Dy > Er > Eu > Yb > Ho > Tb. Ce had the highest content, suggesting that it is the most bioaccumulated element among REEs. The fractionation of REEs in fish was similar to that in Chinese soil [35] (Table 5), indicating that the environmental background plays a dominant role in REE bioaccumulation. The higher content of LREEs compared to HREEs resulted in a ratio commonly exceeding 1, further highlighting the predominance of LREEs in bioaccumulation. As part of the LREEs, the elevated anomalies of Ce and Eu were heavily influenced by the increased aggregation of LREEs in fish, as evidenced by the higher ΔEu and ΔCe values in fish compared to soil. Generally, ΔEu significantly exceeded 1, indicating a clear positive anomaly in Eu among fish, while ΔCe was generally close to 1, indicating a relatively low degree of Ce anomaly among fish.

### 3.2. The Bioaccumulation of REEs in Different Fish Species

**Figure 2:**
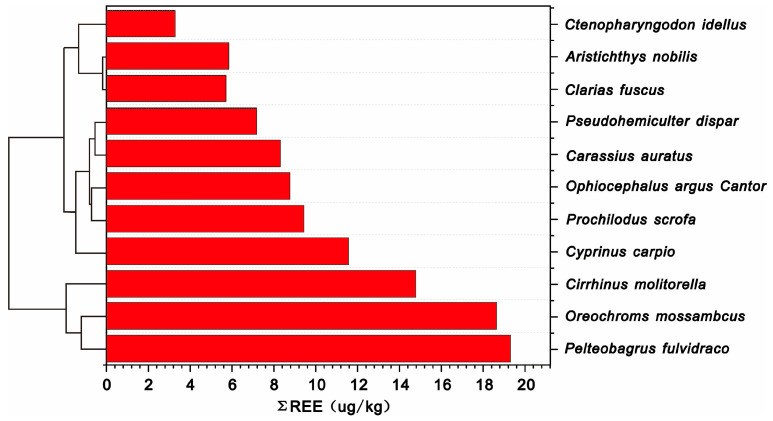
The content of REEs in different fish species.

The total content of REEs in various wild fish species is illustrated in Figure 2, showing a decreasing order of *Pelteobagrus fulvidraco* > *Oreochromis mossambicus* > *Cirrhinus molitorella* > *Cyprinus carpio* > *Prochilodus scrofa* > *Ophiocephalus argus Cantor* > *Carassius auratus* > *Pseudohemiculter dispar* > *Aristichthys nobilis* > *Clarias fuscus* > *Ctenopharyngodon idella*. To further investigate the bioaccumulation of REEs among different fish species, cluster analysis was conducted, and the results are also presented in Figure 2. Based on this analysis, the fish species were divided into three groups. The first group comprises *Aristichthys nobilis*, *Clarias fuscus*, and *Ctenopharyngodon idella*, which have relatively low level of total REEs and should be considered as species with low REEs bioaccumulation. The second group includes *Pelteobagrus fulvidraco*, *Oreochromis mossambicus*, and *Cirrhinus molitorella*, which have significantly higher level of total REEs compared to other species and should be regarded as species with high REE bioaccumulation. The remaining species constitute the third group, including *Cyprinus carpio*, *Prochilodus scrofa*, *Ophiocephalus argus Cantor*, *Carassius auratus*, and *Pseudohemiculter dispar*. The total content of REEs in these species is generally higher than that in the first group but lower than that in the second group, classifying them as species with medium REEs bioaccumulation.

For fractionation of REEs, the LRs/HRs values of species with low bioaccumulation are notably higher than those of medium-bioaccumulation species, and similarly, the LRs/HRs values of medium-bioaccumulation species are significantly higher than those of high-bioaccumulation species (Table 4). This inverse relationship between REEs content and LRs/HRs values indicates that the increase in REE bioaccumulation is not driven by LREEs. Instead, as REEs bioaccumulation increases, it enhances the accumulation of HREEs in fish, thereby reducing the fractionation of REEs within the fish. Regarding ΔEu (europium anomaly), the ΔEu values of species in the high-bioaccumulation group are significantly lower than those in the medium and low-bioaccumulation groups, indicating a decrease in positive Eu anomaly as REEs bioaccumulation increases. In simpler terms, an increase in REEs bioaccumulation can weaken the positive anomaly of Eu. For ΔCe (cerium anomaly), the ΔCe values of species in the medium and high-bioaccumulation groups are generally close to 1, indicating a very low anomaly. In contrast, the ΔCe values of species in the low-bioaccumulation group are significantly below 1, suggesting a clear negative anomaly. This suggests that as REE bioaccumulation increases, the negative anomaly of Ce can be alleviated. Overall, whether considering LRs/HRs, ΔEu, or ΔCe, the fractionation and anomalies of REEs among different fish species decrease as REE bioaccumulation increases.

### 3.3. The Impacts of Feeding Behaviors on REEs Bioaccumulation

Feeding is a pivotal means for fish to ingest contaminants [36]. Fish exhibiting diverse feeding behaviors typically have distinct processes for absorbing contaminants [37], thereby influencing the bioaccumulation of REEs among various fish species (Table 5). Prior research has demonstrated that most contaminants can propagate through the food chain and tend to accumulate in species occupying higher trophic levels [19], leading to generally higher concentrations of various contaminants in carnivorous fish [38]. Conversely, herbivorous fish, which occupy lower trophic levels, exhibit slower bioconversion of most contaminants, resulting in overall lower contaminant levels [39]. The phenomenon of contaminants increasing with trophic level is known as biomagnification. In this study, the species with the lowest accumulation of REEs was found to be *Ctenopharyngodon idellus*, aligning with the overall trend of biomagnification. However, the species with higher REEs bioaccumulation were identified as *Pelteobagrus fulvidraco*, *Oreochromis mossambicus*, and *Cirrhinus molitorella*, all of which are omnivorous rather than carnivorous, yet occupy higher trophic levels. This suggests that the bioaccumulation of REEs is not solely a result of nutrient transfer. Instead, feeding habits also play a role in regulating REEs bioaccumulation in fish. Compared to carnivorous fish, omnivorous fish generally have a broader feeding source. Given that different food sources have varying levels of REEs bioaccumulation, a wider feeding source increases the likelihood of fish being exposed to high-REEs food sources. The duration of this exposure may directly exacerbate its impact on REEs bioaccumulation. Therefore, it is reasonable to believe that a broad feeding source positively influences the increase in REEs bioaccumulation in fish.

For the fractionation of REEs, *Ophiocephalus argus Cantor* and *Clarias fuscus*, being carnivorous species, exhibit relatively higher LRs/HRs, followed by *Ctenopharyngodon idellus*. Conversely, omnivorous species demonstrate lower LRs/HRs among all species. In contrast to LRs/HRs, the ΔCe values of omnivorous species generally approximate 1, indicating no anomaly, whereas those of herbivorous and carnivorous species are notably below 1, indicating a Ce deficiency. Broadly speaking, herbivorous and carnivorous species display higher anomalies, whereas omnivorous species exhibit relatively low anomalies. The minimal fractionation observed in omnivorous species could be attributed to their complex feeding structures. Considering the varied patterns of REEs bioaccumulation across species, the specific feeding approach undoubtedly exacerbates its impact on REEs bioaccumulation in fish. Thus, a diversified diet may hold greater potential to mitigate the influence of a singular feeding approach on REEs bioaccumulation and fractionation. Notably, omnivorous species possess a broader range of food sources compared to herbivorous and carnivorous species, contributing to a wider distribution of trophic level. This variety in feeding sources for omnivorous species prevents excessive impact from specific food sources with high REE fractionation, thereby maintaining an even fractionation of REEs. Conversely, the relatively uniform feeding sources of herbivorous and carnivorous species expose them more frequently to food with higher REE fractionation. The lower LRs/HRs and ΔCe values in omnivorous species closely resemble those of Chinese soil [35], suggesting that omnivorous species are more suitable for indicating environmental REE fractionation than other species.

### 3.4. The Impacts of Living Habitats on REEs Bioaccumulation

The dissolvability of most contaminants is relatively low, allowing only minute quantities to exist in water [40]. Consequently, these contaminants are more prone to existing in particulate form and, under the influence of gravity, aggregating towards the sediments on the riverbed, which serve as a reservoir for numerous contaminants [41]. The extensive accumulation of contaminants in sediments exacerbates their impact on the bioconversion of these pollutants [42]. Given that demersal species have a significantly higher exposure frequency to sediments compared to pelagic species, the bioaccumulation of contaminants in demersal fish is generally higher than in pelagic fish [36], as evidenced in this study. Specifically, the bioaccumulation of REEs is notably higher in demersal species, such as *Pelteobagrus fulvidraco*, *Oreochromis mossambicus*, *Cirrhinus molitorella*, and *Cyprinus carpio*, whereas lower REEs contents are typically found in pelagic species like *Aristichthys nobilis* and *Pseudohemiculter dispa*. This result confirms that, similar to most contaminants, REEs also tend to accumulate more in demersal fish. Although the higher REEs content in demersal fish is a result of the strong stress imparted by sediments, this environmental stress does not aim to diminish the differences between the environmental fractionation of REEs and their fractionation in fish, which is evident from the higher LRs/HRs values observed in most demersal fish. Compared to pelagic fish, the LRs/HRs value of demersal fish deviates further from that found in the environment (soil), with its higher value indicating that the impact of sediments is primarily concentrated in increasing the bioaccumulation of LREEs, while its influence on HREEs bioaccumulation is relatively limited. In fact, previous studies have reported higher toxicity and mutagenicity for HREEs compared to LREEs [43], suggesting that a significant increase in HREEs could pose a significant threat to fish health. In response to the strong stress of REEs in the benthic environment, fish have evolved various strategies, such as altering feeding sources and rates, enhancing the metabolism of HREEs, and shortening lifespans, to avoid the detrimental effects of excessive HREE bioaccumulation [44,45,46]. However, these strategies primarily focus on limiting HREEs bioaccumulation from the benthic environment rather than regulating the bioaccumulation of low-toxicity LREEs. This permissive intake of LREEs exacerbates the fractionation of LREEs and HREEs in demersal fish, increasing the deviation of REEs fractionation between demersal fish and soil. In contrast, the impact of environmental stresses from sediments is limited in surface environments, making it less necessary for pelagic fish to limit their intake of HREEs to maintain health. This unregulated intake of both LREEs and HREEs among pelagic fish evens out their REEs fractionation, making it closer to that found in the environment (soil). Based on our previous analysis, the bioaccumulation of REEs helps to mitigate their anomaly, as seen in demersal fish with strong LREEs bioaccumulation. The lower ΔEu and ΔCe values, closer to 1, indicate a significantly low anomaly in demersal fish. For pelagic fish, their limited bioaccumulation of LREEs makes it difficult to mitigate their REEs anomaly, resulting in higher ΔEu and ΔCe values. The average ΔEu and ΔCe among pelagic fish are 2.64 and above 1, respectively, suggesting a significantly positive anomaly of Eu and a slightly positive anomaly of Ce. In summary, the strong stress from sediments primarily exacerbates the bioaccumulation of LREEs in demersal fish, thereby mitigating their LREEs anomaly.

### 3.5. The Impacts of Fish Size on Their REEs Bioaccumulation

**Figure 3:**
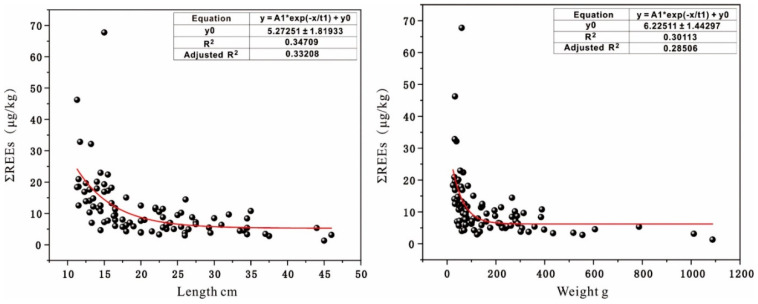
The correlation between fish length, weight, and REEs content in wet weight.

Fish fat primarily consists of carbohydrates, which pollutants seldom penetrate, leading to a common decrease in contaminant bioaccumulation as fat accumulates [21]. Larger fish, with superior physical development, typically have higher fat content compared to smaller fish, resulting in lower contaminant levels [20]. This variation in contaminant content based on fish size is termed biodilution [21]. To assess the impact of biodilution on REEs bioaccumulation, this study analyzed the distribution of REEs in fish of different lengths and weights. Significantly negative correlations were observed between fish length, weight, and their REEs bioaccumulation, confirming that REEs bioaccumulation in fish follows the effect of biodilution (Table 6). While smaller fish generally exhibit higher REEs bioaccumulation, their fractionation cannot match that of larger fish, as evidenced by the positive correlations between fish size and LRs/HRs. This reaffirms the notion that high REEs bioaccumulation aids in mitigating fractionation. However, the differences in REE bioaccumulation among fish of various sizes should be largely attributed to variations in their feeding sources. Previous studies have reported that feeding sources change as fish grow [47,48], primarily due to enhanced predatory abilities associated with physical development. Smaller fish, often physically underdeveloped with limited predatory capabilities, have restricted access to high-energy food sources. To support their rapid growth, they rely on low-energy foods such as phytoplankton, benthic animals and plants, algae, plant debris, organic debris, and humus in sediments, resulting in a complex feeding structure [47,49,50]. In contrast, larger fish possess strong predation abilities and primarily feed on high-energy sources like chironomid larvae, small mollusks, other small fish, shrimp, and benthic insects [47,51]. Since they are in a stable growth phase, they maintain a narrower dietary range, exhibiting a narrower dietary breadth compared to smaller fish. Based on our analysis, a broad feeding range is more likely to enhance REEs bioaccumulation and mitigate fractionation. Although larger fish have a higher feeding energy, their narrow feeding range somewhat limits the influence of feeding on REEs bioaccumulation, leading to stronger REE fractionation. Conversely, the diverse feeding sources of smaller fish not only ensure higher REEs intake but also gradually mitigate fractionation. However, this mitigation does not apply to element anomalies, as evidenced by the correlation between fish size, ΔEu, and ΔCe. There is no significant correlation between fish size and ΔCe, and the correlation with ΔEu is only negative, indicating that Eu anomalies intensify with increasing REEs bioaccumulation in smaller fish. These results suggest that growth-related REEs fractionation in fish is resistant to mitigating element anomalies. To further explore the relationship between fish size and REEs bioaccumulation, a regression analysis was conducted (Figure 3). The relationship between fish length, weight, and REEs content is exponential rather than linear. Fish with high REEs content exceeding 15 μg/kg are primarily found within a range of lengths less than 18 cm and weights below 130 g. Beyond this range, REEs content significantly decreases and remains low. Therefore, fish within these size parameters exhibit higher REE pollution and lower edibility safety, making them suitable as monitoring organisms for REEs pollution in aquatic environments.

### 3.6. The Assessment of Health Risk Associated with REEs Bioaccumulation in Fish

**Figure 4:**
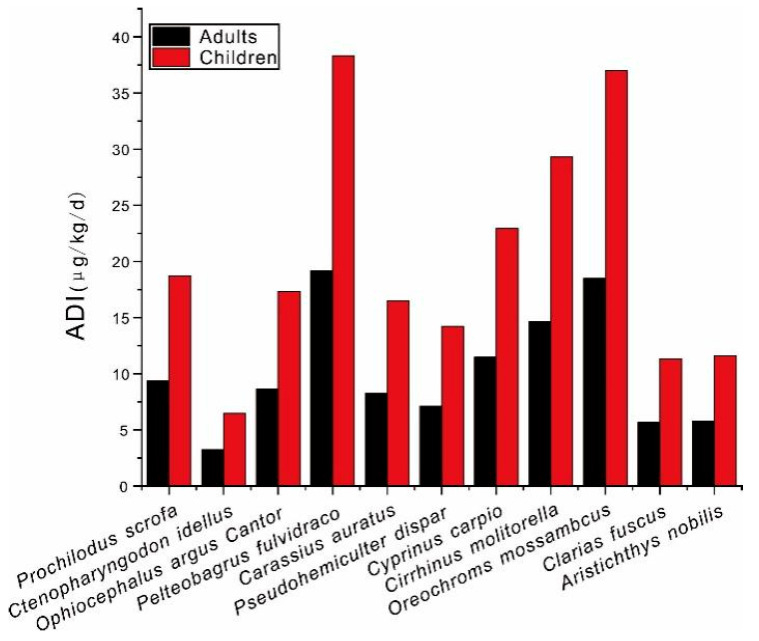
The health risk of different fish species for children and adults.

In order to determine the security of fish consumption, the health risk of REEs bioaccumulation among fish was evaluated in this study (Figure 4). The ADI values of each fish species decreased as following order: *Pelteobagrus fulvidraco* > *Oreochroms mossambcus* > *Cirrhinus molitorella* > *Cyprinus carpio* > *Prochilodus scrofa* > *Ophiocephalus argus Cantor* > *Carassius auratus* > *Pseudohemiculter dispar* > *Aristichthys nobilis* > *Clarias fuscus* > *Ctenopharyngodon idellus*. Among them, the ADI value of Pelteobagrus fulvidraco was found to be highest, which suggested that they hold the maximal risk for public consumption, while the lowest ADI was determined to be *Ctenopharyngodon idellus*, which indicated the minimal risk of their public consumption. Therefore, the introduction of Ctenopharyngodon idellus could be considered as a potential approach to mitigate the health risk of REEs, particularly for the waterways with large emission of REEs.

Due to their smaller body weight, children generally have a lower tolerance for pollutants. In this study, the ADI values of children were significantly higher than those of adults, which once again proves that children face higher health risks from exposure to REEs. Therefore, it is necessary to strengthen the attention to the health risks of REEs in children. According to the report by Ren, Nie, Ma, Wang, Zhao and Liu [34], the threshold for daily intake of ΣREEs is 70 μg/(kg·d). Overall, the ADI values of all fish in this study did not exceed 70 μg/(kg·d), indicating that there are no health risks for fish consumption associated with REEs. However, this does not mean that wild fish in Guangzhou City can be safely consumed with confidence. Due to the concentration of industries and their relevant huge emission of pollution in Guangzhou City, fish are highly likely to be contaminated with other contaminants. Therefore, it is still necessary to strengthen the investigation of health risks among fish that associated with other pollutants. In addition, the centralized industries and their huge emission of REEs in Guangzhou City highlight the key requirement for strengthening environmental biomonitoring, particularly in some hotspots with intense emission of REEs, which is important for pollution control and public health management in the waterways of Guangzhou City.

## 4. Conclusions

The total content of REEs ranged from 1.32 to 67.74 μg/kg, with a predominant presence of light REEs. The ΔEu and ΔCe values, which exceeded and approached 1, respectively, indicated positive Eu anomalies and low Ce anomalies. Wild fish were categorized into high-, medium-, and low-REEs-bioaccumulation groups using cluster analysis. Higher LRs/HRs and ΔEu values, coupled with lower ΔCe values, in fish from the high-bioaccumulation group suggested that increased bioaccumulation mitigated fractionation. Omnivorous fish with higher REEs levels and lower LRs/HRs indicated that broader feeding sources may enhance REE bioaccumulation and diminish fractionation. Elevated REE concentrations and LRs/HRs in demersal fish highlighted a preferential accumulation of light REEs in the benthic environment. Smaller fish with higher REEs levels but lower LRs/HRs were likely associated with complex feeding sources. Regression analysis revealed that fish with lengths and weights of less than 18 cm and 130 g, respectively, were more susceptible to REEs bioaccumulation. Despite higher ADI values indicating a greater risk for children and *Pelteobagrus fulvidraco*, all ADI values within 70 μg/(kg·d) suggested that fish consumption poses no risk. This study confirmed that the fractionation of REEs in fish can be used to trace their bioconversion.

## Figures and Tables

**Table 1:** Detailed information of the fish samples.

Species	Num	Length	Weight	Living Habitats	Feeding Behaviors
		cm	g		
*Prochilodus scrofa*	8	23–27	176.6–287.9	Demersal	Omnivore
*Ctenopharyngodon idellus*	3	18.2–22.5	90.1–127.1	Demersal	Herbivore
*Ophiocephalus argus Cantor*	8	26.5–34.5	163.5–434.3	Demersal	Carnivore
*Pelteobagrus fulvidraco*	3	12.1–15.2	24.7–31.6	Demersal	Omnivore
*Carassius auratus*	11	11.5–23	223.8	Demersal	Omnivore
*Pseudohemiculter dispar*	10	13–20	19–60.4	Pelagic	Omnivore
*Cyprinus carpio*	30	13–26.5	30.1–606.1	Demersal	Omnivore
*Cirrhinus molitorella*	18	16.5–46	56.7–1087.6	Demersal	Omnivore
*Oreochroms mossambcus*	35	11.3–26	31.3–334.3	Demersal	Omnivore
*Clarias fuscus*	5	22–30	102.6–265.8	Demersal	Carnivore
*Aristichthys nobilis*	6	21.5–44	110.7–786.5	Pelagic	Omnivore

**Table 2:** The operating conditions of ICP-MS.

ICP-MS	
RF power	1550 W
Plasma, carrier and makeup gas flow	14, 1.0025 and 0.8 L min^−1^, respectively
Spray chamber temperature	2 °C
Cones	Pt
Integration time	0.1 s
Peak pattern	1 point per mass
Replicates per analysis	10

**Table 3:** The relevant parameters for health risk assessment.

Parameters	Children	Adults	References
GW (g/d)	59.69	59.69	[20,34]
EF (days/year)	365	365	
ED (year)	6	70	
BW (kg)	30	60	
AT (day/year)	6 × 365	70 × 365	

Note: The average body weight was only considered for Chinese people in this study.

**Table 4:** Total content and fractionation of rare earth elements in fish (μg/kg wet weight).

	REEs	LRs/HRs	ΔEu	ΔCe	LREEs	HREEs
All fish	13.14 ± 11.43	4.99 ± 2.06	1.85 ± 0.94	1.02 ± 0.18	10.64 ± 9.29	2.50 ± 2.28
Chinese Soil [35]	176.75	3.78	0.65	0.96	139.79	36.96
*Prochilodus scrofa*	9.42 ± 6.05	4.62 ± 1.1	1.26	1.04 ± 0.13	7.65 ± 4.91	1.76 ± 1.17
*Ctenopharyngodon idellus*	3.26	6.75	—	0.74	2.84	0.42
*Ophiocephalus argus Cantor*	8.72 ± 6.95	7.01 ± 1.36	—	0.93 ± 0.19	7.73 ± 6.35	0.99 ± 0.6
*Pelteobagrus fulvidraco*	19.29	4.25	1.26	1.05	15.62	3.67
*Carassius auratus*	8.30 ± 4.69	5.94 ± 2.24	2.32 ± 0.77	1.00 ± 0.18	6.89 ± 3.81	1.41 ± 0.9
*Pseudohemiculter dispar*	7.16 ± 3.15	4.43 ± 0.57	—	1.24	5.76 ± 2.42	1.40 ± 0.73
*Cyprinus carpio*	11.55 ± 5.82	5.63 ± 2.41	2.22 ± 0.89	1.01 ± 1.04	9.70 ± 5.08	1.86 ± 0.96
*Cirrhinus molitorella*	14.75 ± 15.66	5.51 ± 1.45	1.68 ± 1.00	1.07 ± 0.29	12.37 ± 13.11	2.38 ± 2.59
*Oreochroms mossambcus*	18.62 ± 13.53	3.45 ± 1.23	1.63 ± 0.87	1.03 ± 0.1	14.28 ± 10.9	4.34 ± 2.92
*Clarias fuscus*	5.71 ± 2.76	7.21 ± 2.79	—	0.85 ± 0.02	4.79 ± 2.12	0.91 ± 0.65
*Aristichthys nobilis*	5.84 ± 2.85	5.33 ± 1.04	2.64 ± 0.45	0.98 ± 0.19	4.90 ± 2.43	0.94 ± 0.44

Note: LRs/HRs is the ratio between light REEs and heavy REEs. The *p* values of REEs, LREEs, and HREEs between species were found to be lower than 0.05, which suggested their significant difference, while those of other parameters were all higher than 0.05, expressing non-significant difference.

**Table 5:** The bioaccumulation and fractionation of REEs in fish with different feeding behaviors.

	ΣREEs	LRs/HRs	ΔEu	ΔCe	LREEs	HREEs
Omnivore	11.86	4.90	1.86	1.05	9.65	2.22
Carnivore	7.22	7.11	—	0.89	6.26	0.95
Herbivore	3.26	6.75	—	0.74	2.84	0.42
Pelagic	6.50	4.88	2.64	1.11	5.33	1.17
Demersal	11.07	5.60	1.73	0.97	9.10	1.97
Chinese Soil	176.75	3.78	0.65	0.96	139.79	36.96

Note: The *p* values of REEs, ΔEu, LREEs, and HREEs between species with different feeding behaviors were found to be lower than 0.05, which suggested their significant difference, while those of other parameters were all higher than 0.05, expressing non-significant difference. The *p* values of fish with different living habitats were all found to be lower than 0.05, which showed their non-significant difference.

**Table 6:** The correlation between fish length, weight, REEs bioaccumulation, and fractionation.

	Weight	ΣREEs	LRs/HRs	ΔEu	ΔCe
Length	0.920 *	−0.500 *	0.418 *	−0.470 *	0.019
Weight	-	−0.392 *	0.290 *	−0.441 *	−0.043

* Correlation is significant at the 0.01 level (2-tailed).

## Data Availability

The datasets used and/or analyzed during the current study are available from Xiongyi Miao on reasonable request.

## References

[1] Han R., Zhang Q., Wang D., Zhong Q., Han G. (2025). Discrimination of brewing technologies and assessment of health risks based on rare earth elements: Evidence of fingerprint in Chinese famous vinegars. Food Chem..

[2] Siddique N., Chaudhary M.Z., Anjum M., Abid J. (2024). Pollution level assessment, source apportionment, and health hazards of heavy metals and rare earth elements in the sediment core from the coast of Karachi, Pakistan. Mar. Pollut. Bull..

[3] Wang W., Huang X., Chen S., Li L., Wang Y., Kang Y., Nie Y. (2024). Geochemical characteristics of sediments in the southern Mid-Atlantic Ridge indicate hydrothermal activity: Evidence from rare earth elements. Mar. Pet. Geol..

[4] Bi D., Shi X., Huang M., Shen F., Yu M., Zhang Y., Shi F., Liu J. (2024). Enhanced deep-water circulation facilitated rare earth elements enrichment in pelagic sediments from the northwestern Pacific Ocean. Glob. Planet. Change.

[5] Gwenzi W., Mangori L., Danha C., Chaukura N., Dunjana N., Sanganyado E. (2018). Sources, behaviour, and environmental and human health risks of high-technology rare earth elements as emerging contaminants. Sci. Total Environ..

[6] Diao H., Yang H., Tan T., Ren G., You M., Wu L., Yang M., Bai Y., Xia S., Song S. (2024). Navigating the rare earth elements landscape: Challenges, innovations, and sustainability. Miner. Eng..

[7] He M., Liu G., Li Y., Zhou L., Wang G., Si W., Xie Z. (2024). Rare earth elements in the upstream of Yangtze River Delta: Spatio-temporal distributions, sources and speciations. Mar. Pollut. Bull..

[8] Ma S., Han G. (2024). Rare earth elements reveal the human health and environmental concerns in the largest tributary of the Mekong river, Northeastern Thailand. Environ. Res..

[9] Gu Y.-G., Wang Y.-S., Jordan R.W., Su H., Jiang S.-J. (2023). Probabilistic ecotoxicological risk assessment of heavy metal and rare earth element mixtures in aquatic biota using the DGT technique in coastal sediments. Chemosphere.

[10] Bancel S., Cachot J., Bon C., Rochard É., Geffard O. (2024). A critical review of pollution active biomonitoring using sentinel fish: Challenges and opportunities. Environ. Pollut..

[11] Hao Y., Wei X., Zhao X., Zhang X., Cai J., Song Z., Liao X., Chen X., Miao X. (2024). Bioaccumulation, contamination and health risks of trace elements in wild fish in Chongqing City, China: A consumer guidance regarding fish size. Environ. Geochem. Health.

[12] Khaled R., Elabed S., Masarani A., Almulla A., Almheiri S., Koniyath R., Semerjian L., Abass K. (2023). Human biomonitoring of environmental contaminants in Gulf Countries—Current status and future directions. Environ. Res..

[13] Hsu W.-H., Zheng Y., Sanghamitra S.S., Liu M., Elizabeth L.L.-M., Kenneth M.A., Parsons P.J., Kannan K., Rej R., Wang W. (2022). Biomonitoring of exposure to Great Lakes contaminants among licensed anglers and Burmese refugees in Western New York: Toxic metals and persistent organic pollutants, 2010–2015. Int. J. Hyg. Environ. Health.

[14] Ali M.U., Wang C., Li Y., Jin X., Yang S., Ding L., Feng L., Wang B., Li P. (2023). Human biomonitoring of heavy metals exposure in different age- and gender-groups based on fish consumption patterns in typical coastal cities of China. Ecotoxicol. Environ. Saf..

[15] Miao X., Zhang Q., Hao Y., Zhang H. (2023). The Size Screening Could Greatly Degrade the Health Risk of Fish Consuming Associated to Metals Pollution—An Investigation of Angling Fish in Guangzhou, China. Toxics.

[16] Recabarren-Villalón T., Ronda A.C., Oliva A.L., Cazorla A.L., Marcovecchio J.E., Arias A.H. (2021). Seasonal distribution pattern and bioaccumulation of Polycyclic aromatic hydrocarbons (PAHs) in four bioindicator coastal fishes of Argentina. Environ. Pollut..

[17] Yu X., Gutang Q., Wang Y., Wang S., Li Y., Li Y., Liu W., Wang X. (2024). Microplastic and associated emerging contaminants in marine fish from the South China Sea: Exposure and human risks. J. Hazard. Mater..

[18] Kumar M., Singh S., Jain A., Yadav S., Dubey A., Trivedi S.P. (2024). A review on heavy metal-induced toxicity in fishes: Bioaccumulation, antioxidant defense system, histopathological manifestations, and transcriptional profiling of genes. J. Trace Elem. Med. Biol..

[19] Hao Y., Miao X., Song M., Zhang H. (2022). The Bioaccumulation and Health Risk Assessment of Metals among Two Most Consumed Species of Angling Fish (*Cyprinus carpio* and *Pseudohemiculter dispar*) in Liuzhou (China): Winter Should Be Treated as a Suitable Season for Fish Angling. Int. J. Environ. Res. Public Health.

[20] Miao X., Hao Y., Tang X., Xie Z., Liu L., Luo S., Huang Q., Zou S., Zhang C., Li J. (2020). Analysis and health risk assessment of toxic and essential elements of the wild fish caught by anglers in Liuzhou as a large industrial city of China. Chemosphere.

[21] Li J., Miao X., Hao Y., Xie Z., Zou S., Zhou C. (2020). Health Risk Assessment of Metals (Cu, Pb, Zn, Cr, Cd, As, Hg, Se) in Angling Fish with Different Lengths Collected from Liuzhou, China. Int. J. Environ. Res. Public Health.

[22] Lin H., Zhou L., Lu S., Yang H., Li Y., Yang X. (2024). Occurrence and spatiotemporal distribution of natural and synthetic steroid hormones in soil, water, and sediment systems in suburban agricultural area of Guangzhou City, China. J. Hazard. Mater..

[23] Liu Z., Rong H., Chu Z., Luo H., Zhao M., Wang R., Zhang C. (2021). Screening and quantification of pharmaceuticals and their metabolites in municipal wastewater treatment facilities in Guangzhou, China. Desalination Water Treat..

[24] Huang L., Liu D., Cai D.-j., Chen S.-j., Dong H., Lin G.-z., Wang B.-g., Yang J. (2022). Health risk assessment of exposure to multiple pollutants in Guangzhou. China Environ. Sci..

[25] Ma L., Wang W.-X. (2023). Dissolved rare earth elements in the Pearl River Delta: Using Gd as a tracer of anthropogenic activity from river towards the sea. Sci. Total Environ..

[26] Ma L., Dang D.H., Wang W., Evans R.D., Wang W.-X. (2019). Rare earth elements in the Pearl River Delta of China: Potential impacts of the REE industry on water, suspended particles and oysters. Environ. Pollut..

[27] Wang S., Lin C., Cao X. (2011). Heavy metals content and distribution in the surface sediments of the Guangzhou section of the Pearl River, Southern China. Environ. Earth Sci..

[28] Wang S., Cao X., Lin C., Chen X. (2010). Arsenic content and fractionation in the surface sediments of the Guangzhou section of the Pearl River in Southern China. J. Hazard. Mater..

[29] Qing L., Xiaojuan H., Jiangluan J., Junyi Z., Zhihui W., Yufeng Y. (2014). Comparison of the water quality of the surface microlayer and subsurface water in the Guangzhou segment of the Pearl River, China. J. Geogr. Sci..

[30] Zhao Y., Wu R., Cui J., Gan S., Pan J., Guo P. (2020). Improvement of water quality in the Pearl River Estuary, China: A long-term (2008–2017) case study of temporal-spatial variation, source identification and ecological risk of heavy metals in surface water of Guangzhou. Environ. Sci. Pollut. Res..

[31] National Sharing Platform for Reference Materials. https://www.ncrm.org.cn/Web/OrderingEn/MaterialDetail?autoID=8116.

[32] Wang X.-N., Gu Y.-G., Wang Z.-H. (2022). Rare earth elements in different trophic level marine wild fish species. Environ. Pollut..

[33] USEPA (2020). Integrated Risk Information System (IRIS).

[34] Ren L., Nie H., Ma W., Wang L., Zhao W., Liu Y. (2024). Concentration, correlation, and health risk assessment of rare earth elements in different edible parts of the swimming crab (*Portunus trituberculatus*) in Shandong Province, China. Mar. Pollut. Bull..

[35] Wei F., Liu Y., Teng E., Rui K. (1991). Background value characteristics of rare earth elements in Chinese soil. Environ. Sci..

[36] Jiang X., Wang J., Pan B., Li D., Wang Y., Liu X. (2022). Assessment of heavy metal accumulation in freshwater fish of Dongting Lake, China: Effects of feeding habits, habitat preferences and body size. J. Environ. Sci..

[37] Garneroa P.L., Monferran M.V., González G.A., Griboff J., de los Ángeles B.M. (2018). Assessment of exposure to metals, As and Se in water and sediment of a freshwater reservoir and their bioaccumulation in fish species of different feeding and habitat preferences. Ecotoxicol. Environ. Saf..

[38] Gall J.E., Boyd R.S., Rajakaruna N. (2015). Transfer of heavy metals through terrestrial food webs: A review. Environ. Monit. Assess..

[39] Gao S., Zhang R., Zhang H., Zhang S. (2022). The seasonal variation in heavy metal accumulation in the food web in the coastal waters of Jiangsu based on carbon and nitrogen isotope technology. Environ. Pollut..

[40] Miao X., Song M., Xu G., Hao Y., Zhang H. (2022). The Accumulation and Transformation of Heavy Metals in Sediments of Liujiang River Basin in Southern China and Their Threatening on Water Security. Int. J. Environ. Res. Public Health.

[41] Miao X., Chen L., Hao Y., An J., Xu T., Bao W., Chen X., Liao X., Xie Y. (2024). The variations of heavy metals sources varied their aggregated concentration and health risk in sediments of karst rivers—A case study in Liujiang River Basin, Southwest China. Mar. Pollut. Bull..

[42] Miao X., Hao Y., Liu H., Xie Z., Miao D., He X. (2021). Effects of heavy metals speciations in sediments on their bioaccumulation in wild fish in rivers in Liuzhou—A typical karst catchment in southwest China. Ecotoxicol. Environ. Saf..

[43] Sysolyatina M.A., Olkova A.S. (2023). Sources of rare earth elements in the environment and their impact on living organisms. Environ. Rev..

[44] Porras-Rivera G., Górski K., Colin N. (2024). Behavioral biomarkers in fishes: A non-lethal approach to assess the effects of chemical pollution on freshwater ecosystems. Environ. Res..

[45] Shahjahan M., Taslima K., Rahman M.S., Al-Emran M., Alam S.I., Faggio C. (2022). Effects of heavy metals on fish physiology—A review. Chemosphere.

[46] Hasan A.K.M.M., Hamed M., Hasan J., Martyniuk C.J., Niyogi S., Chivers D.P. (2024). A review of the neurobehavioural, physiological, and reproductive toxicity of microplastics in fishes. Ecotoxicol. Environ. Saf..

[47] Croizier G.L., Schaal G., Gallon R., Fall M., Grand F.L., Munaron J.-M., Rouget M.-L., Machu E., Loc’h F.L., Laë R. (2016). Trophic ecology influence on metal bioaccumulation in marine fish: Inference from stable isotope and fatty acid analyses. Sci. Total Environ..

[48] Yang B., Qu X., Liu H., Yang M., Xin W., Wang W., Chen Y. (2024). Urbanization reduces fish taxonomic and functional diversity while increases phylogenetic diversity in subtropical rivers. Sci. Total Environ..

[49] Arcagni M., Juncos R., Rizzo A., Pavlin M., Fajon V., Arribére M.A., Horvat M., Ribeiro S.G. (2017). Species- and habitat-specific bioaccumulation of total mercury and methylmercury in the food web of a deep oligotrophic lake. Sci. Total Environ..

[50] Soares J.M., Gomes J.M., Reis G.C.L., Hoyos D.C.M., Custódio F.B., Gloria M.B.A. (2021). Biogenic amines in amazonian fish and their health effects are affected by species and season of capture. Food Control.

[51] Kuipers R.S., Luxwolda M.F., Offringa P.J., Boersma E.R., Dijck-Brouwer D.A.J., Muskieta F.A.J. (2012). Gestational age dependent changes of the fetal brain, liver and adipose tissue fatty acid compositions in a population with high fish intakes. Prostaglandins Leukot. Essent. Fat. Acids.

